# Supplementary material for: Do age, gender, and education modify the effectiveness of app-delivered and tailored self-management support among adults with low back pain?—Secondary analysis of the selfBACK randomised controlled trial
Source: PLOS Digit Health. 2023 Sep 22;2(9):e0000302. doi: 10.1371/journal.pdig.0000302 (PMC10516425; doi:10.1371/journal.pdig.0000302)
Supplement: S1 Table — (DOCX) [file pdig.0000302.s002.docx]

S1 Table: Population characteristics across sociodemographic variables for intervention and control group

|  | Age | | | | | | Gender | | | | Education | | | |
| --- | --- | --- | --- | --- | --- | --- | --- | --- | --- | --- | --- | --- | --- | --- |
|  | 18-34 years (n=103) | | 35-64 years (n=295) | | ≥65 years  (n=63) | | Male  (n=206) | | Female  (n=255) | | ≤12 years  (n=164) | | >12 years  (n=297) | |
|  | UC (n=51) | SB (n=52) | UC  (n=153) | SB  (n=142) | UC  (n=25) | SB  (n=38) | UC  (n=95) | SB  (n=111) | UC  (n=134) | SB  (n=121) | UC  (n=84) | SB (n=80) | UC (n=145) | SB  (n=152) |
| Country of recruitment |  |  |  |  |  |  |  |  |  |  |  |  |  |  |
| Norway, % | 58 | 42 | 45 | 55 | 0 | 100 | 40 | 60 | 56 | 44 | 50 | 50 | 48 | 52 |
| Denmark, % | 40 | 60 | 55 | 45 | 43 | 57 | 49 | 51 | 51 | 49 | 51 | 49 | 49 | 51 |
| Recruitment site, (%) |  |  |  |  |  |  |  |  |  |  |  |  |  |  |
| Physiotherapy | 53 | 47 | 50 | 50 | 44 | 56 | 47 | 53 | 52 | 48 | 49 | 51 | 50 | 50 |
| Chiropractor | 38 | 62 | 67 | 43 | 29 | 71 | 49 | 51 | 50 | 50 | 54 | 47 | 47 | 53 |
| General practice | 68 | 32 | 42 | 58 | 0 | 100 | 38 | 62 | 57 | 43 | 31 | 69 | 55 | 45 |
| SpineCentre | 44 | 56 | 53 | 47 | 43 | 57 | 45 | 55 | 54 | 46 | 57 | 43 | 44 | 56 |
| Age (years), mean | 26.9 | 27.9 | 49.5 | 49.7 | 70.2 | 70.6 | 46.8 | 48.0 | 46.7 | 48.5 | 53.1 | 53.8 | 43.0 | 45.3 |
| Female, % | 38 | 62 | 52 | 28 | 33 | 67 | 0 | 0 | 46 | 54 | 60 | 40 | 49 | 51 |
| LBP, average last week, NRS | 4,8 | 4.7 | 4.9 | 4.9 | 4.9 | 4.7 | 4.8 | 5.0 | 5.0 | 4.7 | 5.2 | 5.2 | 4.8 | 4.7 |
| LBP, duration >12 weeks, % | 54 | 46 | 52 | 48 | 42 | 58 | 46 | 54 | 53 | 47 | 50 | 50 | 49 | 51 |

Abbreviations: no. = number, LBP = low back pain.

*Question only asked to those reporting full-or part time employment.
