# Supplementary material for: Do age, gender, and education modify the effectiveness of app-delivered and tailored self-management support among adults with low back pain?—Secondary analysis of the selfBACK randomised controlled trial
Source: PLOS Digit Health. 2023 Sep 22;2(9):e0000302. doi: 10.1371/journal.pdig.0000302 (PMC10516425; doi:10.1371/journal.pdig.0000302)
Supplement: S2 Table — (DOCX) [file pdig.0000302.s003.docx]

S2 Table: Mean and difference between groups at three and nine months for Roland Morris Disability Questionnaire.

|  | |  | Mean (SD)^a^ | | | | |
| --- | --- | --- | --- | --- | --- | --- | --- |
|  | | n | Baseline | 6 wks | 3 mths | 6 mths | 9 mths |
| Age | |  |  |  |  |  |  |
| Age 18-34 years | Usual care | 51 | 10.1 (4.3) | 6.7 (5.2) | 6.6 (5.9) | 5.2 (5.3) | 4.4 (4.7) |
|  | selfBACK | 52 |  | 6.3 (4.3) | 5.7 (4.4) | 4.6 (5.1) | 5.4 (5.9) |
| Age 35-64 years | Usual care | 153 | 10.5 (4.4) | 7.8 (5.4) | 7.4 (5.4) | 7.2 (5.3) | 7.1 (5.6) |
|  | selfBACK | 142 |  | 7.4 (4.6) | 6.8 (4.8) | 6.3 (4.9) | 6.2 (5.2) |
| Age ≥65 years | Usual care | 25 | 10.7 (4.5) | 9.4 (4.6) | 9.1 (4.5) | 9.9 (5.2) | 9.9 (5.5) |
|  | selfBACK | 38 |  | 8.0 (5.0) | 7.4 (4.5) | 7.2 (5.5) | 6.2 (5.1) |
| Gender |  |  |  |  |  |  |  |
| Male | Usual care | 95 | 10.7 (4.4) | 7.4 (5.7) | 7.3 (5.9) | 6.7 (6.1) | 6.6 (5.2) |
|  | selfBACK | 111 |  | 7.7 (4.7) | 6.9 (4.8) | 6.1 (5.6) | 5.8 (5.2) |
| Female | Usual care | 134 | 10.2 (4.4) | 8.0 (5.0) | 7.5 (5.1) | 7.3 (5.0) | 7.0 (5.2) |
|  | selfBACK | 121 |  | 6.9 (4.5) | 6.5 (4.7) | 6.1 (4.5) | 6.3 (5.3) |
| Education | |  |  |  |  |  |  |
| ≤ 12 years | Usual care | 84 | 10.6 (4.5) | 8.6 (5.3) | 8.6 (5.0) | 7.7 (5.4) | 7.6 (5.7) |
|  | selfBACK | 80 |  | 7.9 (4.0) | 7.6 (4.5) | 6.8 (4.9) | 6.4 (5.6) |
| > 12 years | Usual care | 145 | 10.4 (4.3) | 7.3 (5.2) | 6.7 (5.6) | 6.7 (5.4) | 6.4 (5.6) |
|  | selfBACK | 152 |  | 7.0 (4.9) | 6.2 (4.8) | 5.7 (5.1) | 5.8 (5.1) |

Abbreviations: SD = standard deviation

^a^Marginal means from a crude linear mixed model, and SDs from raw data among persons with information at the specific time points
