# Supplementary material for: Do age, gender, and education modify the effectiveness of app-delivered and tailored self-management support among adults with low back pain?—Secondary analysis of the selfBACK randomised controlled trial
Source: PLOS Digit Health. 2023 Sep 22;2(9):e0000302. doi: 10.1371/journal.pdig.0000302 (PMC10516425; doi:10.1371/journal.pdig.0000302)
Supplement: S3 Table — (DOCX) [file pdig.0000302.s004.docx]

S3 Table: Mean and difference between groups at three and nine months for LBP intensity.

|  | |  | Mean (SD)^a^ | | | | |
| --- | --- | --- | --- | --- | --- | --- | --- |
|  | | n | Baseline | 6 wks | 3 mths | 6 mths | 9 mths |
| Age | |  |  |  |  |  |  |
| Age 18-34 years | Usual care | 51 | 4.7 (2.0) | 4.0 (2.3) | 3.8 (2.3) | 3.2 (2.0) | 3.1 (2.4) |
|  | selfBACK | 52 |  | 3.2 (2.3) | 3.1 (1.9) | 2.9 (2.1) | 2.7 (2.5) |
| Age 35-64 years | Usual care | 153 | 4.9 (1.9) | 4.1 (2.2) | 4.1 (2.5) | 4.0 (2.4) | 3.9 (2.4) |
|  | selfBACK | 142 |  | 3.6 (2.2) | 3.3 (2.3) | 3.1 (2.3) | 3.2 (2.3) |
| Age ≥65 years | Usual care | 25 | 5.0 (1.8) | 4.2 (1.7) | 3.2 (1.9) | 4.8 (2.6) | 3.9 (2.4) |
|  | selfBACK | 38 |  | 4.0 (2.4) | 3.3 (2.2) | 3.3 (2.0) | 3.0 (2.1) |
| Gender |  |  |  |  |  |  |  |
| Male | Usual care | 95 | 4.9 (1.9) | 3.9 (2.2) | 3.8 (2.3) | 3.8 (2.4) | 3.7 (2.5) |
|  | selfBACK | 111 |  | 3.5 (2.4) | 3.4 (2.4) | 3.2 (2.5) | 3.0 (2.3) |
| Female | Usual care | 134 | 4.9 (1.9) | 4.2 (2.1) | 4.0 (2.4) | 4.0 (2.3) | 3.8 (2.3) |
|  | selfBACK | 121 |  | 3.6 (2.2) | 3.1 (1.9) | 3.0 (1.9) | 3.1 (2.2) |
| Education | |  |  |  |  |  |  |
| ≤ 12 years | Usual care | 84 | 5.2 (1.8) | 4.7 (2.2) | 4.3 (2.3) | 4.3 (2.5) | 4.3 (2.4) |
|  | selfBACK | 80 |  | 4.0 (2.4) | 3.7 (2.4) | 3.4 (2.4) | 3.3 (2.4) |
| > 12 years | Usual care | 145 | 4.7 (2.0) | 3.7 (2.1) | 3.7 (2.4) | 3.7 (2.3) | 3.4 (2.3) |
|  | selfBACK | 152 |  | 3.3 (2.2) | 3.1 (2.0) | 3.0 (2.1) | 2.9 (2.2) |

Abbreviations: SD = standard deviation

^a^Marginal means from a crude linear mixed model, and SDs from raw data among persons with information at the specific time points
