# Supplementary material for: Do age, gender, and education modify the effectiveness of app-delivered and tailored self-management support among adults with low back pain?—Secondary analysis of the selfBACK randomised controlled trial
Source: PLOS Digit Health. 2023 Sep 22;2(9):e0000302. doi: 10.1371/journal.pdig.0000302 (PMC10516425; doi:10.1371/journal.pdig.0000302)
Supplement: S4 Table — (DOCX) [file pdig.0000302.s005.docx]

S4 Table: Mean and difference between groups at three and nine months for Pain Self-Efficacy Questionnaire.

|  | |  | Mean (SD)^a^ | | | | |
| --- | --- | --- | --- | --- | --- | --- | --- |
|  | | n. | Baseline | 6 wks | 3 mths | 6 mths | 9 mths |
| Age | |  |  |  |  |  |  |
| Age 18-34 years | Usual care | 51 | 44.8 (9.0) | 47.5 (10.7) | 46.6 (10.4) | 48.9 (10.3) | 49.7 (10.2) |
|  | selfBACK | 52 |  | 48.9 (8.8) | 48.4 (8.8) | 49.8 (10.7) | 50.4 (8.6) |
| Age 35-64 years | Usual care | 153 | 43.5 (11.8) | 45.1 (12.5) | 46.3 (11.7) | 46.4 (11.6) | 46.1 (11.2) |
|  | selfBACK | 142 |  | 47.7 (10.2) | 49.2 (10.6) | 49.5 (10.5) | 50.0 (10.0) |
| Age ≥65 years | Usual care | 25 | 45.6 (10.4) | 44.8 (12.4) | 47.9 (10.0) | 46.3 (9.6) | 45.9 (10.3) |
|  | selfBACK | 38 |  | 48.1 (9.1) | 50.2 (10.0) | 49.4 (10.3) | 51.0 (10.1) |
| Gender |  |  |  |  |  |  |  |
| Male | Usual care | 95 | 44.0 (10.6) | 46.8 (12.6) | 47.7 (10.2) | 47.8 (10.4) | 47.3 (11.4) |
|  | selfBACK | 111 |  | 47.9 (9.2) | 48.3 (9.8) | 49.0 (11.4) | 51.0 (8.8) |
| Female | Usual care | 134 | 44.2 (11.4) | 44.7 (11.5) | 45.9 (11.7) | 46.4 (11.6) | 46.6 (10.7) |
|  | selfBACK | 121 |  | 48.0 (10.1) | 50.0 (10.0) | 50.1 (9.3) | 49.5 (10.4) |
| Education | |  |  |  |  |  |  |
| ≤ 12 years | Usual care | 84 | 42.0 (11.5) | 43.3 (12.8) | 44.3 (10.7) | 45.3 (10.6) | 44.6 (10.6) |
|  | selfBACK | 80 |  | 46.9 (9.3) | 48.1 (11.0) | 48.6 (11.5) | 48.2 (10.8) |
| > 12 years | Usual care | 145 | 45.2 (10.6) | 46.9 (11.5) | 47.8 (11.3) | 47.9 (11.5) | 48.1 (11.1) |
|  | selfBACK | 152 |  | 48.5 (9.9) | 49.8 (9.3) | 50.1 (9.8) | 51.3 (8.8) |

Abbreviations: SD = standard deviation

^a^Marginal means from a crude linear mixed model, and SDs from raw data among persons with information at the specific time points
