# Supplementary material for: Do age, gender, and education modify the effectiveness of app-delivered and tailored self-management support among adults with low back pain?—Secondary analysis of the selfBACK randomised controlled trial
Source: PLOS Digit Health. 2023 Sep 22;2(9):e0000302. doi: 10.1371/journal.pdig.0000302 (PMC10516425; doi:10.1371/journal.pdig.0000302)
Supplement: Attachment — Submitted filename: REVIEWER REMARKS FOR MANUSCRIPT PDIG-D-22-00340.pdf [file pdig.0000302.s006.pdf]

REVIEWER REMARKS FOR MANUSCRIPT PDIG-D-22-00340

1. **Summary**

The paper is looking at an important topic of research focusing on application of Artificial Intelligence based self-management support for Lower Back Pain(LBP).The gist of the work is to establish whether the variables age, gender and education are effect modifiers of the digital intervention that has been proposed. The methodology adopted is secondary analysis of randomized control trial involving 461 study subjects recruited from across Demark and Norway. The participants were randomized into two treatment arms : the control group had 229 subjects who received the usual care , the treatment arm (232 subjects) received the digital intervention in addition to the usual care. RMDQ scores were taken at baseline, week six, three , six and nine months. Stratified analysis was applied. Main finding indicate that the variables age, gender and education have no modifier effect on the effectiveness of the digital intervention outcome variable (LBP)-related disability. However, it was noted that the intervention had a positive effect on the older study subjects, in the long run, compared to the younger participants.

2. **Overall Impression**

The research is of great relevance. The study has been well undertaken. The methodology is sound. The analysis is adequate.

3. **Major Issue(s)**

a) The title of the work appear to be unclear. This is because the main objective of the work was to determine if the sociodemographic variables (age, gender, education) had modifier effect on the effectiveness of the intervention.

b) Can the authours clarify if the “ low back pain” captured on the title mean LBD-related disability or is it consisting of both the primary outcome and the two secondary outcomes.

c) There appears to be three (3) response variables in this work: LBD-related disability, LBD intensity and pain self-efficacy. Only the primary outcome (LBD-related disability) has been analyzed. It would be good if the authours can explain why the other two outcomes have not been considered in detail.

d) Can the authours explain how the sample size of 461 was arrived at.

4. **Minor issue(s)**

a) In Lines 195 and 248 reference is made to manuscripts under submission or review. Can this be captured differently just in case the authours’ manuscript comes out earlier than the two references.
