# Supplementary material for: Do age, gender, and education modify the effectiveness of app-delivered and tailored self-management support among adults with low back pain?—Secondary analysis of the selfBACK randomised controlled trial
Source: PLOS Digit Health. 2023 Sep 22;2(9):e0000302. doi: 10.1371/journal.pdig.0000302 (PMC10516425; doi:10.1371/journal.pdig.0000302)
Supplement: Attachment — Submitted filename: Response to Reviewers.docx [file pdig.0000302.s007.docx]

# Response to reviewers

Dear Editor,

We thank the editor and the reviewers for the evaluation and the opportunity to further improve the manuscript. The manuscript has been revised in accordance with the editor and reviewers’ comments and suggestions.

Below is a point-by-point reply to all comments from the reviewers. All changes in the manuscript are marked with tracked changes.

# Reviewer #1:

The authors examined the effect modification of the promising digital health intervention app, the selfBACK, and clearly showed that pain-related disability was not modified by age, gender, education. However, this work still contains some inadequacies in the data analysis and discussions. Although I understand that it is unrealistic to acquire new clinical data because this study is a secondary analysis of the previous randomized controlled trial, I listed some issues that need to be addressed in order to improve this work mature enough for publication in this journal.

**1**.	Figure 1A and table 2 indicate that there is a tendency for the selfBACK intervention to be more effective in the elderly and less effective in the rest of the population, even though without any significance. Could it be a reason for the results of the RCT that is mentioned as “the effect was as small and of uncertain clinical significance” at line 66? The authors should discuss this possibility to try to explain about the uncertainty in the RCT.

**Authors reply:** Thank you for a relevant comment. We discussed your suggestion in the author group and have given it a lot of consideration. We do however feel that it would be problematic to use the results from this secondary analysis as a standalone to explain the uncertainties in the RCT. As we argue in the discussion, the results on effect modification should be interpreted with caution since the power calculations is based on the main RCT and additional analysis of effect modification may increase the likelihood of chance findings in these secondary analyses. In addition, the oldest group in the present secondary analysis included only 63 participants, and thus the statistical uncertainty of the effect estimate within this subgroup is substantial.

**Revisions:** We have added a sentence in the discussion to emphasise that the statistical uncertainty of the effect estimate within the oldest subgroup is substantial (Page 10, line 198-199).

**2.**	Do the authors have the detailed information about what kind of usual care the participants took at each time point of the RCT? If they do, they should clearly show the information for both the intervention group and the control group to help readers further understand the effect modification of the selfBACK.

**Authors reply:** Unfortunately, we do not have detailed information about health care use during the follow-up at this time point. The participants were informed that the app was a supplement to their usual care and that they should follow any advice given by their clinician. Monitoring of the actual treatments or advice was not a part of the protocol for the RCT. Possible synergistic effect between self-management support and usual care can therefore not be excluded. This was also discussed as a limitation of the original RCT (2021). The selfBACK trial also included a process evaluation with interviews of participants and clinicians. These data are now being analyzed and will be published in a separate paper. The data from the process evaluation may provide valuable insights into the content of usual care for selected groups of participants.

**Revisions:** We have added a sentence in the method-section to clarify that the participants were informed that the app was a supplement to their usual care and that they should follow any advice given by their clinician (Page 5, line 114-115).

We have also added in the discussion that a possible synergistic effect between self-management support and usual care cannot be excluded (Page 10, line 205-208).

*Sandal LF, Bach K, Overas CK, Svendsen MJ, Dalager T, Stejnicher Drongstrup Jensen J, et al. Effectiveness of App-Delivered, Tailored Self-management Support for Adults With Lower Back Pain-Related Disability: A selfBACK Randomized Clinical Trial. JAMA Intern Med. 2021;181(10):1288-96.*

**3.**	This work including the previous RCT does not seem to have shown how AI-based tailored support improved LBP, because it might be still possible that the usage of a digital tool without AI in itself could encourage the LBP self-management enough. Nevertheless, at line 250-251, the authors mentioned “we have so far not found any consistent effect modification related to the selfBACK intervention, which may be due to the individual tailoring of the intervention.” This sentence could lead to a biased discussion. If the authors use the word “AI-based” in the title of this article, they should show or discuss whether it is the AI-based tailored support or just a support by a digital tool that improved the LBP self-management.

**Authors reply:** We agree that future studies should investigate whether AI-based tailoring introduce additional effects beyond non-tailored digital support. However, this was not the primary aim of the RCT (or this secondary analysis). The design does not allow us to show the comparison suggested by the reviewer. We have therefore deliberately used the phrasing “…, which may be due to…” to emphasize that this is speculative. Further, the mentioning of a possible effect of the individual approach does not necessarily mean AI-based tailoring.

**Revisions:** We have added a sentence to the discussion identifying the need for further studies investigating whether AI-based tailoring introduce additional beneficial effects compared to non-tailored digital support (Page 13, line 268-269)

4.	The author should clarify what kind of future research plans they need to proceed to based on the results of this secondary analysis, which readers might strongly want to know for further development of this promising app.

**Authors reply:** The results of this and the other secondary analysis from the RCT alongside the results on the process evaluation performed in parallel to the RCT will all be published and shared with the scientific community to inspire future research. Currently, the cost-effectiveness of the app is being investigated in the Danish cohort, which may inform the further development of the app and implementation in clinical practice.

**Revisions**: we have added a sentence to the discussion to clarify the future plans for obtaining information informing the app development (page 13, line 269-271)

<Minor points>

I listed below some points which might be typos. Please correct each of them, if it should be corrected.

1.	Both “SELFBACK” and “selfBACK” can be seen through the whole texts. Which is correct?

**Authors reply**: Thank you for pointing out this inconsistency. The correct is ‘selfBACK’ (small caps) and this has been corrected throughout.

2.	At line 167: “35-64 years” ---> a typo for “18-34 years” ?

**Authors reply** Thank you; this certainly is a typo. This has now been corrected in the manuscript (Page 7, Line 177)

3.	In table 2: With mean difference between genders for RMDQ,

“-0.4” at 3 months ---> “0.4” ?

“0.4” at 9 months ---> “-0.4” ?

**Authors reply:** We thank the reviewer for guiding us to check these numbers. They are actually both correct. However, when checking we found a typo in the 95% CI. This should be -1.8 to 1.1 and not -0.8 to 1.1. This has been corrected in the manuscript.

4.	In table 3: With mean difference between genders for LBP intensity,

“-0.4” at 3 months ---> “0.4” ?

“0.3” at 9 months ---> “-0.3” ?

**Authors reply:** We have checked this again, and the numbers given in table 3 are correct.

5.	In table 4: With mean difference between genders for Pain Self-Efficacy Questionnaire,

“3.2” at 3 months ---> “-3.2” ?

“-0.5” at 9 months ---> “1.0” ?

**Authors reply:** We have checked this again, and the numbers given in table 4 are correct.

**Revision**: We have added a sentence to the table legends to support the reader in interpretation of the mean difference in effect due to interaction in tables 2-4.

# Reviewer #2:

The manuscript is technically sound. The objective is well framed. The methodology to address the objective is well covered. Explanation has been given of how the study participants were recruited. The study design (RCT) is appropriate as the interest is on the treatment effect. Ethical consideration has been captured. The conclusions are based on the findings from the study using the data that was collected. The statistical analysis is appropriate though a linear model with the treatment, age, gender and education as independent variables could have been explored. All the data have been made available. The manuscript is well structured and written in standard English

**Summary**

The paper is looking at an important topic of research focusing on application of Artificial Intelligence based self-management support for Lower Back Pain(LBP).The gist of the work is to establish whether the variables age, gender and education are effect modifiers of the digital intervention that has been proposed. The methodology adopted is secondary analysis of randomized control trial involving 461 study subjects recruited from across Demark and Norway. The participants were randomized into two treatment arms: the control group had 229 subjects who received the usual care , the treatment arm (232 subjects) received the digital intervention in addition to the usual care. RMDQ scores were taken at baseline, week six, three , six and nine months. Stratified analysis was applied. Main finding indicate that the variables age, gender and education have no modifier effect on the effectiveness of the digital intervention outcome variable (LBP)-related disability. However, it was noted that the intervention had a positive effect on the older study subjects, in the long run, compared to the younger participants.

**Overall Impression**

The research is of great relevance. The study has been well undertaken. The methodology is sound. The analysis is adequate.

**Major Issue(s)**

1.	The title of the work appear to be unclear. This is because the main objective of the work was to determine if the sociodemographic variables (age, gender, education) had modifier effect on the effectiveness of the intervention.

**Authors reply:** We do agree that the title is a bit unclear in several aspects and have revised it to better clarify the purpose of the paper.

**Revisions:** We have now changed the title to: *Do age, gender and education modify the effectiveness of app-delivered and tailored self-management support among adults with low back pain? - Secondary analysis of the selfBACK randomised controlled trial* (Page 1, line 1-3)

2.	Can the authors clarify if the “ low back pain” captured on the title mean LBD-related disability or is it consisting of both the primary outcome and the two secondary outcomes.

**Authors reply:** “Low back pain” as captured in the title was meant to capture all the outcomes (both primary and secondary). However, we see that this is not clearly explained. As you so correctly pointed out in your previous comment, the title was unclear in several aspects. By rephrasing the title, we now use the term “low back pain” to describe the target group and not the outcomes. We hope this will clarify the purpose of the paper. 3.	There appears to be three (3) response variables in this work: LBD-related disability, LBD intensity and pain self-efficacy. Only the primary outcome (LBD-related disability) has been analyzed. It would be good if the authours can explain why the other two outcomes have not been considered in detail.

**Authors reply:** The reviewer is correct that the manuscript concerns three outcomes: LBP-related disability, LBP intensity and pain self-efficacy. They are all reported in the manuscript (see figure 1 and tables 2-4). The results for all three outcomes are summarized in the second paragraph in the results section (page 7, lines 174-175), “*We observed no effect modification by gender, or education at any time-point or for any outcome (tables 2-4)*”.

4.	Can the authours explain how the sample size of 461 was arrived at.

**Authors reply:** The sample size was determined for the selfBACK RCT and has been explained in detail in the protocol paper. We acknowledge that this was not clear in the manuscript and we have added a sentence to accommodate this (Page 6, Line 145—146)

*Sandal LF et al. An App-Delivered Self-Management Program for People With Low Back Pain: Protocol for the selfBACK Randomized Controlled Trial. JMIR Res Protoc. Dec 3 2019;8(12):e14720. doi:10.2196/14720*

**Minor issue(s)**

1.	In Lines 195 and 248 reference is made to manuscripts under submission or review. Can this be captured differently just in case the authours’ manuscript comes out earlier than the two references.

**Authors reply:** In line 195 and 248 we refer to the same paper. We agree that it is problematic to refer to papers that are not accepted for publication. However, we choose to do so because these two parallel papers are highly relevant for each other. Of course, we cannot be sure about the timing of publications, but the paper we are referring to is now just waiting for editorial decision. Thus, we prefer to keep the reference as it is and will include the full reference if the paper is published before the current paper. We will remove it in proof reading if this is not the case.

**Revision:** We refer to the paper as Nordstoga et al (Manuscript in revision) in the text and include the paper in the reference list with the same status. (reference 31)

# Reviewer #3:

Overall, the manuscript is very well written and clear. I only have some very minor comments:

1.	Some sentences could be added in the introduction section on how this work extends the previous works on this app by the authors. The contributions of this work compared to the four past papers by the team could be elaborated.

**Authors reply:** Thank you for your kind comments. We agree that the link between the previous works from the selfBACK study and the present study is not clearly presented in the introduction.

**Revisions:** We have now included some sentences in the introduction clarifying how the present study adds to the papers of Sandal et al (2021), Øverås et al (2022) and Rughani et al (2023). (Page 3, Line 67-72)

*Sandal LF, Bach K, Overas CK, Svendsen MJ, Dalager T, Stejnicher Drongstrup Jensen J, et al. Effectiveness of App-Delivered, Tailored Self-management Support for Adults With Lower Back Pain-Related Disability: A selfBACK Randomized Clinical Trial. JAMA Intern Med. 2021;181(10):1288-96.*

*Overas CK, Nilsen TIL, Nicholl BI, Rughani G, Wood K, Sogaard K, et al. Multimorbidity and co-occurring musculoskeletal pain do not modify the effect of the SELFBACK app on low back pain-related disability. BMC Med. 2022;20(1):53.*

*Rughani G, Nilsen TIL, Wood K, Mair FS, Hartvigsen J, Mork PJ, Nicholl BI. The selfBACK artificial intelligence-based smartphone app can improve low back pain outcome even in patients with high levels of depression or stress. Eur J Pain. 2023; 27(5):568-579.*

2.	How does lower back pain monitoring impact workplace productivity? Is there any study or future scope to study how these participants manage their pain while at the workplace? This could be discussed in the discussion section, for example, on the use of your app outdoors or at work. There are some recent apps and devices that are applicable for self-monitoring at the workplace. A review paper is here for reference: <https://doi.org/10.1002/aisy.202100099>, V.B. Patel et al, Advanced Intelligent Systems, 4: 2100099, 2022

**Authors reply:** We thank the reviewer for bringing this point forward. A separate analysis is underway examining if their occupational status and level of physical activity at work modify the effect of the selfBACK app. However, we do not have data to support an analysis on how monitoring LBP symptoms may affect work productivity or how the participants manage their LBP while at work. Thus, we feel that a discussion of this issue is beyond the scope of our paper.
